# A new system for quantitative evaluation of infant gaze capabilities in a wide visual field

**DOI:** 10.1186/s12938-015-0076-7

**Published:** 2015-09-07

**Authors:** Andrea Pratesi, Francesca Cecchi, Elena Beani, Giuseppina Sgandurra, Giovanni Cioni, Cecilia Laschi, Paolo Dario

**Affiliations:** The BioRobotics Institute, Scuola Superiore Sant′Anna, Viale Rinaldo Piaggio 34, 56025 Pontedera, Pisa Italy; Department of Developmental Neuroscience, IRCCS Fondazione Stella Maris, Viale del Tirreno 331, 56128 Calambrone, Pisa Italy; Department of Neuroscience, Psychology, Drug Research and Child Health, University of Florence, Florence, Italy; Department of Clinical and Experimental Medicine, University of Pisa, Via Roma 67, 56125 Pisa, Italy

**Keywords:** Eye-tracker, Gaze-tracking, Infant gaze, Neurodevelopmental bioengineering

## Abstract

**Background:**

The visual assessment of infants poses specific challenges: many techniques that are used on adults are based on the patient’s response, and are not suitable for infants. Significant advances in the eye-tracking have made this assessment of infant visual capabilities easier, however, eye-tracking still requires the subject’s collaboration, in most cases and thus limiting the application in infant research. Moreover, there is a lack of transferability to clinical practice, and thus it emerges the need for a new tool to measure the paradigms and explore the most common visual competences in a wide visual field. This work presents the design, development and preliminary testing of a new system for measuring infant’s gaze in the wide visual field called CareToy C: CareToy for Clinics.

**Methods:**

The system is based on a commercial eye tracker (SmartEye) with six cameras running at 60 Hz, suitable for measuring an infant’s gaze. In order to stimulate the infant visually and audibly, a mechanical structure has been designed to support five speakers and five screens at a specific distance (60 cm) and angle: one in the centre, two on the right-hand side and two on the left (at 30° and 60° respectively). Different tasks have been designed in order to evaluate the system capability to assess the infant’s gaze movements during different conditions (such as gap, overlap or audio-visual paradigms). Nine healthy infants aged 4–10 months were assessed as they performed the visual tasks at random.

**Results:**

We developed a system able to measure infant’s gaze in a wide visual field covering a total visual range of ±60° from the centre with an intermediate evaluation at ±30°. Moreover, the same system, thanks to different integrated software, was able to provide different visual paradigms (as gap, overlap and audio-visual) assessing and comparing different visual and multisensory sub-competencies. The proposed system endowed the integration of a commercial eye-tracker into a purposive setup in a smart and innovative way.

**Conclusions:**

The proposed system is suitable for measuring and evaluating infant’s gaze capabilities in a wide visual field, in order to provide quantitative data that can enrich the clinical assessment.

## Background

The assessment of visual capabilities in the first year of life is important for monitoring the development of the infant, but also because if problems occur, they can consequently influence the whole development [1, 2]. One goal of child health development screening programs is to identify, as early as possible, infants at risk of future visual problems or, more generally, developmental disabilities, in order to make an early intervention possible and incisive [3]. Visual assessments of infants are not easy: many assessment techniques that are used with adults are based on the patient’s response thus not suitable for infants since they have such a short attention span. Nevertheless, in recent decades it has become possible to use new assessment methods that have been adapted for the needs of younger patients, that require neither the collaboration of the subject nor special abilities of the examiner; so they are suitable for very young infants, non-verbal or non-collaborative subjects (e.g. patient with mental delay or behavioural problems).

In recent years, eye-tracking has become an increasingly popular tool among researchers in the neurodevelopmental field [4]. Significant advances, particularly in the area of automated eye-tracking, have made this technology much more user-friendly and applicable to human infant populations than ever before [5]. Traditionally, recording eye movements in infants was difficult because of the small field of view required for accurate capture of the pupil. During the test, if the infant steps out from the camera view, data was lost and, of course, infants do not follow instructions to sit still. Other systems such as chinrests and head-mounted optics (cameras and other components) commonly used with adult participants are again impractical for infants since they are more invasive. However, there has been significant progress in the production of lightweight models appropriate for infants. Despite this fast growing interest in infant eye-tracking, the use of this technology has been mostly applied to capturing infant eye movements and gaze patterns when looking at objects or scenes depicted in two dimensions on a computer screen [6–11]. In the studies mentioned above, visual stimulations appear on screens that represent the central visual field and gaze movements are mainly made by the contribution of the eyes. In fact, gaze (the direction of the visual axis in space) is the sum of two contributors: the eye position relative to the head and the head position relative to the space. Over the past few years, eye tracking has been used to examine a variety of perceptual and cognitive phenomena, including categorisation [6, 7, 12–21]. Very few attempts have been developed to assess infant eye tracking in the context of multisensory stimulation in a wide working field covering the peripheral visual field. These studies reported the use of head mounted eye-trackers. These devices require participants to either wear a cap or a band placed on the crown of their head, or wear goggles resting in front of their eyes [5]. Although these devices allowed the participants to navigate in 3D space, several drawbacks such as the invasiveness, the data accuracy in 3D space and the difficulties in detecting eye and head contributions limit their application to infant research. From this analysis, the lack of transferability to clinical practice emerges, and thus the need for a new tool to measure the paradigms and explore the most common visual competences in a wide visual field. This need as a starting point, the aim of this work was to design, develop and test a new system called CareToy C: CareToy for Clinics for quantitatively measuring infant’s gaze in a wide visual field (120°) during different conditions (such as gap, overlap or audio-visual paradigms). The innovation was to develop a single system for providing different paradigms and to be able to measure and compare infants’ eye movements. This work has been inserted into the framework of the EU CareToy project.

## Methods

On the basis of the clinical requirements, the following features have to be taken into account. The system should be able to:

- measure wide range of visual field (120°);
- provide audio-visual stimulation;
- identify five different areas of the visual field for audio-visual stimulation covering 120°;
- measure infants gaze i.e. single contribution of eyes and head respectively;
- represent a non intrusive setup and an ecological approach during clinical trials;
- be adapted according to the infants’ needs (aged 3–12 months).

### Hardware

**Fig. 1:**
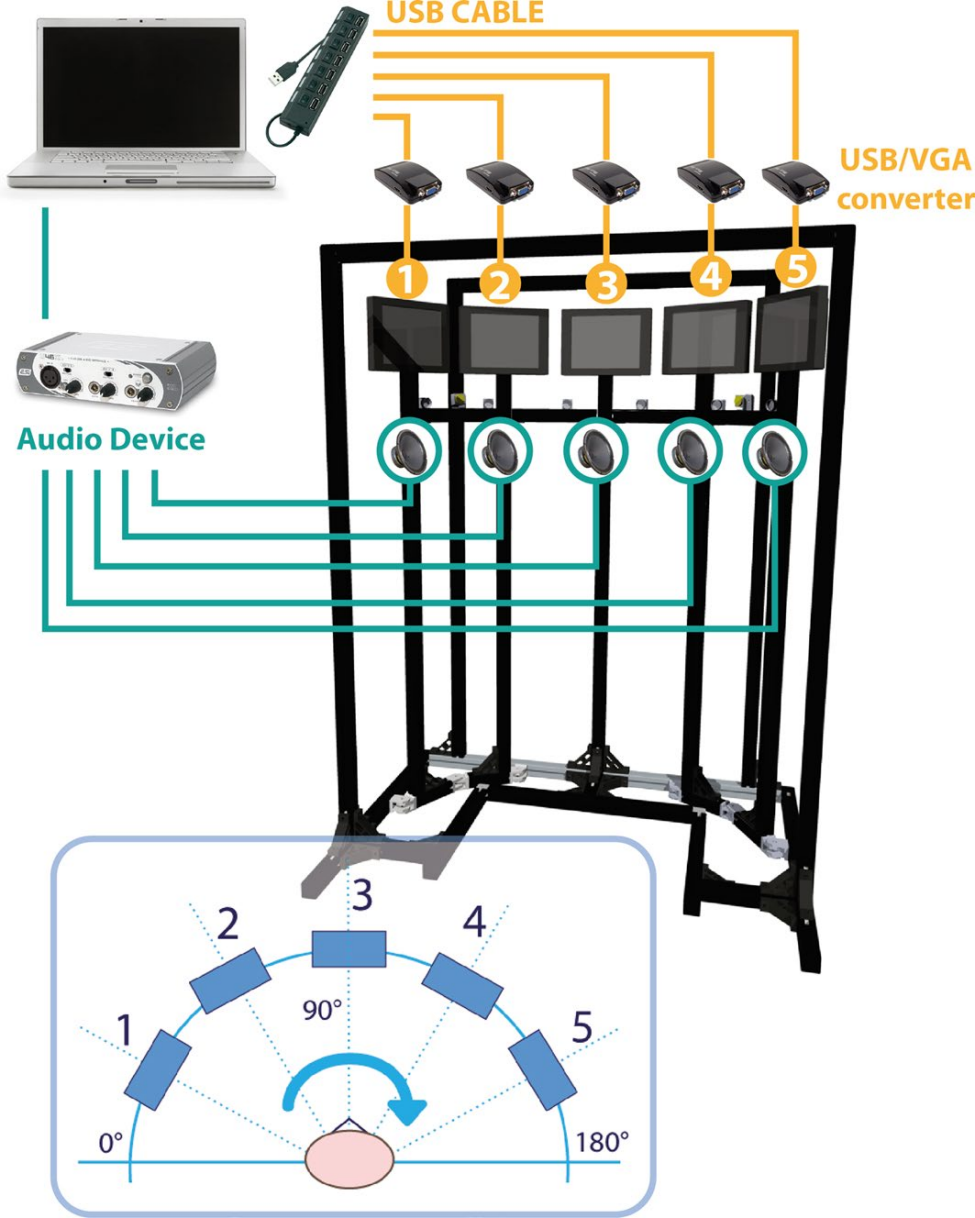
Schematic system overview. The mechanical custom structure represents the support for the five screens, the five speakers, the six SmartEye cameras running at 60 Hz and two IR-diodes used for illuminating the face of the subject in order to minimize the effect of varying environmental lighting conditions and for using the reflections of these IR flashes on the cornea (“glints”) to find the centre of the eyes. The stimuli management has been obtained using a laptop combined with the audio–video external devices. In the *lower part* of this overview, it is possible to observe the gaze heading frame of reference

The system consists of an eye-tracker integrated into a customised mechanical structure. The five points are placed in specific positions: one in the centre, two on the right side (30° and 60°) and two on the left side (30° and 60°). In order to provide audio-visual stimulations, each point of interest comprises a screen (10.5″) and a speaker. A mechanical structure has been designed in order to fix the screens and the speakers at specific distances and angles (Fig. 1). The screens active area represents our area of interest (AOI), so our stimuli dimensions are 220 × 129 mm.

We selected USB-VGA converters and an external audio device for managing multi-monitors images and sounds signals. Custom software has been developed for the management of the audio-visual stimulations. A dedicated PC has been assigned for the management of these audio-visual stimuli. The external sound card device with six outputs is used to send the sound to the five speakers; the sound card communicates with the PC via the USB port and is connected to the speakers with RCA cables. The desktop is extended to five screens using the USB/VGA converters (Fig. 1).

As far as the eye trackers are concerned, the technological background of the eye tracking technology has been studied to choose the best solutions for our application. In order to obtain a non-intrusive system for measuring the infant gaze, video-oculography (VOG) has been selected. This family of eye trackers is based on corneal-reflection. They assess a video input of the pupil’s highlight reflected on the cornea, usually from a light source invisible to humans, in the infrared range of the spectrum. The centre of the pupil and the corneal reflection are tracked in real time, providing information about the participant’s point of gaze (POG) on the stimulus [4]. Among the available eye trackers we selected the SmartEye system with six cameras running at 60 Hz due to the following main features:

*Most flexible cameras placement in our setup* One of the main advantages of the SmartEye system is its flexibility. The cameras can be positioned independently one from the other, but each camera should be oriented in such way so that the subject’s head is the camera’s point of focus. In addition, the number of cameras is not fixed but can be adapted in order to cover the required visual field (±60°).

**Fig. 2:**
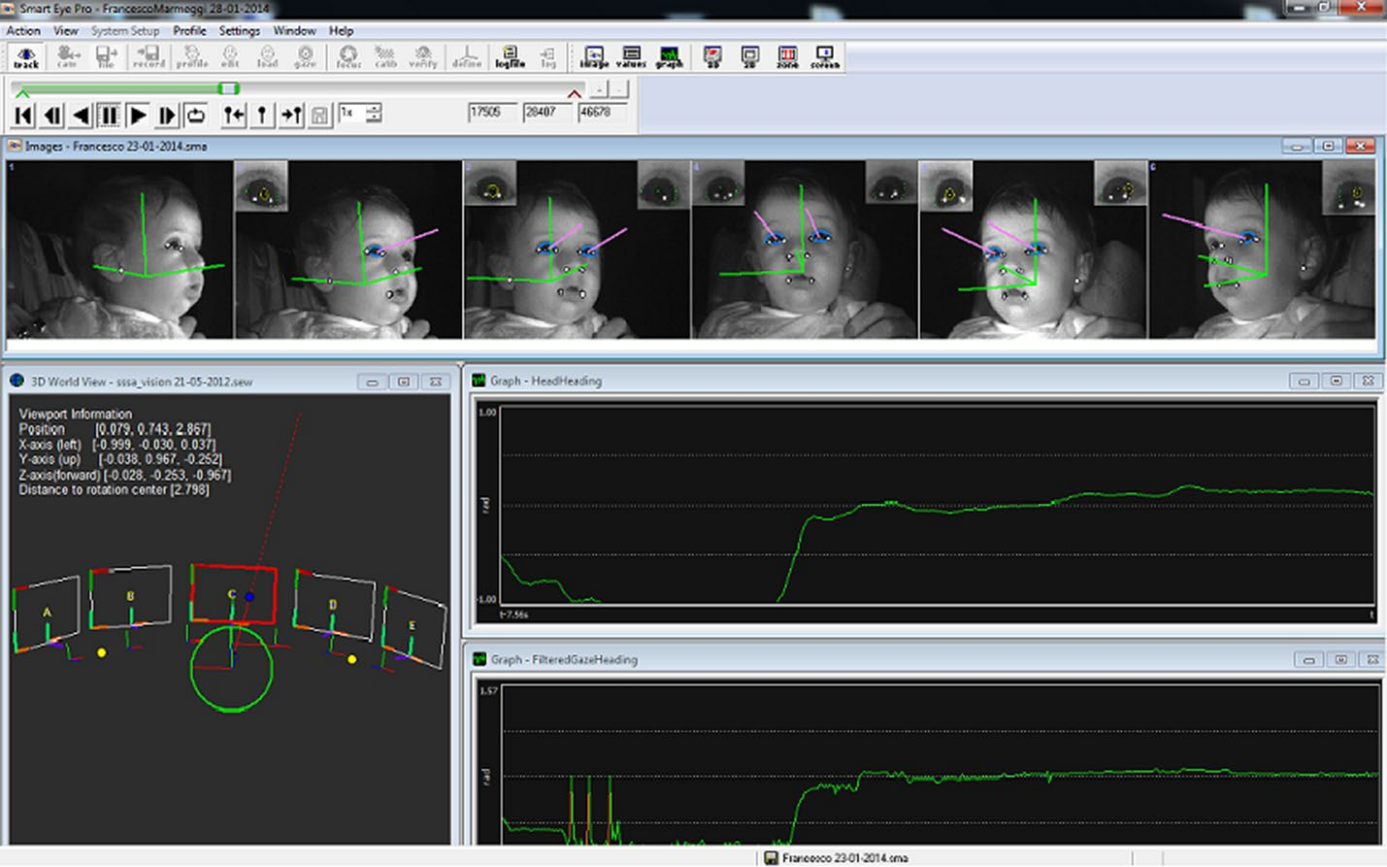
SmartEye Graphical User Interface (GUI). In the *upper bar* it is possible see the *pink vectors* that represent the infant’s gaze vector; in the *lower* window on the *left*, there is the 3D representation of the external 3D setup with the visualisation of gaze intersection on a object modelled in the 3D world (i.e. the gaze vector intersects screen n.3) and on the *right* there are the typical gaze heading and head heading signal profiles

**Fig. 3:**
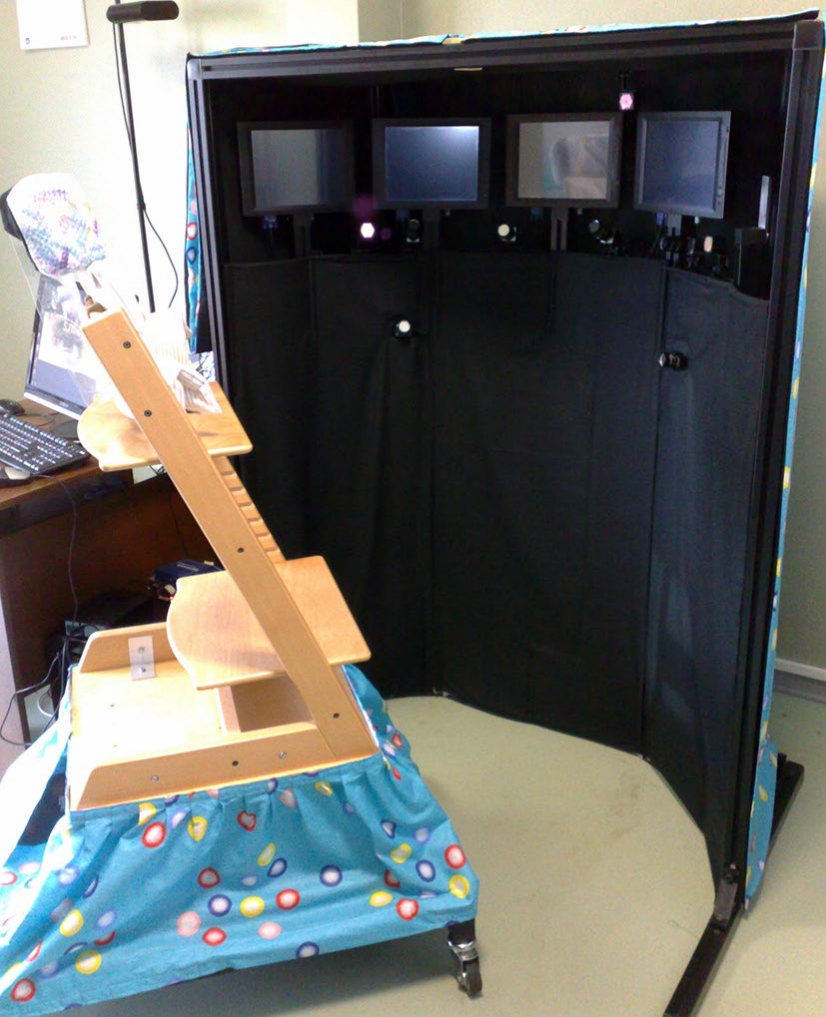
The final version of the system

*Gaze and head tracking* The system uses IR-diodes to illuminate the face of the subject in order to minimize the effect of varying lighting conditions and use the reflections of these IR flashes on the cornea (“glints”) to find the centre of the eyes, rather than other systems where the eye centre is estimated using the head model (Fig. 2). This allows a more accurate identification of gaze direction with fewer errors in the head pose estimation. This feature, i.e. the possibility to measure both eye and head components is extremely important since the visual field proposed in this work is wide and each movement of the infant to reach the audio-visual stimulus is composed of both components. Finally, in order to complete the system and to adjust the setup according to the infant’s needs, a seat has been purposely designed allowing for adjustments to the height (the infant’s eyes should be at the height of the screen’s centre) and the distance from the screen (≈60 cm). In addition, two different accessories complete the seat that allow us to position infants that have not yet reached the stage of torso control, as well as infants that can maintain the sitting posture independently (Fig. 3).

### Software

Purposive software has been developed to create sequences of stimuli (images and sounds) and send them to the screens and speakers. Through the GUI it is possible to set some parameters: (a) the total number of images composing the audio-visual tasks sequences; (b) the stimuli (images and sounds) to be presented on each screen and (c) the duration of each stimulus (each sound and image can have a different duration). SmartEye System computer desktop and the PC that manages the presentation of the stimuli are connected to a Local Area Network (LAN) and they synchronize their local time using the Network Time Protocol (NTP).

### Experiments

#### Clinical background and hypotheses

Focusing on the assessment of visual attention, the disengagement of attention is the most studied function (engagement, disengagement and attention shift) by using gap and/or overlap paradigms [22, 23]. In both cases there are two visual stimuli, one on the midline and the other one the on periphery. Both paradigms study the infant’s gaze shift from the central to the peripheral stimulus. In the gap condition, the midline stimulus disappears before the peripheral one appears, while it remains present in the overlap paradigm. Clinical studies, without using an eye-tracker, have shown that infants in their first year of life need more time to shift the attention from a central to a peripheral stimulus in the overlap condition [24–26]. Moreover, this effect is more evident in younger infants and decreases with age; this allows us to hypothesize a maturational effect on the gaze shifting competence.

Another interesting aspect is the association between visual and auditory stimuli. When they are combined, the response latencies are fastest with audio-visual targets than the visual targets alone (slower), or the auditory targets (slowest) [27]. All the paradigms have been applied in a limited visual field.

We expected that the purposive setup composing the CareToy C and the specific software for the management of the audiovisual stimuli would allow the clinicians to develop clinical tasks for the quantitative assessment of visual attention i.e. time and degree of gaze (head and eyes components, separately) in all three (gap, overlap and multisensory) paradigms in a wide field condition.

#### Experimental paradigm

The infant is placed at 60 cm from the screens and the environment is black to avoid distracting factors and to allow the infant to devote their attention to the audio-visual stimuli. The stimuli were chosen in order to be interesting and attractive for infants in their first year of life. There are two series of images for two main categories of age: geometric, circular, concentric or high contrasted pictures (like black/white chessboards, concentric black, white and red images) for younger infants and human faces for older infants. We designed three different tasks to test our hypotheses:

- “attention task” (AT) is the reproduction of the gap paradigm: the stimulus is presented on the central screen; after 3 s this stimulus disappears and a different stimulus is proposed on one of the peripheral screens (30° or 60° on the left/right side, randomly); we expected that the system would be able to measure the shifts of gaze in a non-competitive situation both at 30° and 60° on the right and on the left side of visual field.
- “fixation task” (FT), is the reproduction of the overlap paradigm: the stimulus is presented on the central screen; after 3 s a lateral stimulus simultaneously appears on the peripheral screen (30° or 60° on the left/right side, randomly) in competition with the central one; we expected that the system would be able to measure the shifts of gaze in a competitive situation both at 30° and 60° on the right and on the left side of visual field.
- “audio-visual task” (AVT), the stimulus is presented on the central screen; after 3 s an audio stimulus is presented simultaneously with a visual one on the peripheral screen (i.e. spatially and temporary coherent); we expected that the system would be able to simultaneously produce the audio and visual stimuli and that it would be able to measure the same parameters of AT, detecting the differences in the time parameters between the two tasks (AT and AVT).

The basic sequences of each task were repeated in order to obtain a global duration of 100–120 s. These durations were calibrated based on the average time of visual attention in the first year of life.

#### Sample

Nine healthy, born at term, infants (4 males, 5 females) with an age range between 4 and 10 months (mean 7 ± 1.73 months) were assessed using this system at the IRCCS Fondazione Stella Maris. Each infant performed three different tasks described below (attention, fixation and audio-visual) in a random order. This clinical trial has been approved by the Ethics Committee of Pisa University Hospital and the Tuscan Region Pediatric Ethics Committee (Italy).

### Parameters of interest

**Fig. 4:**
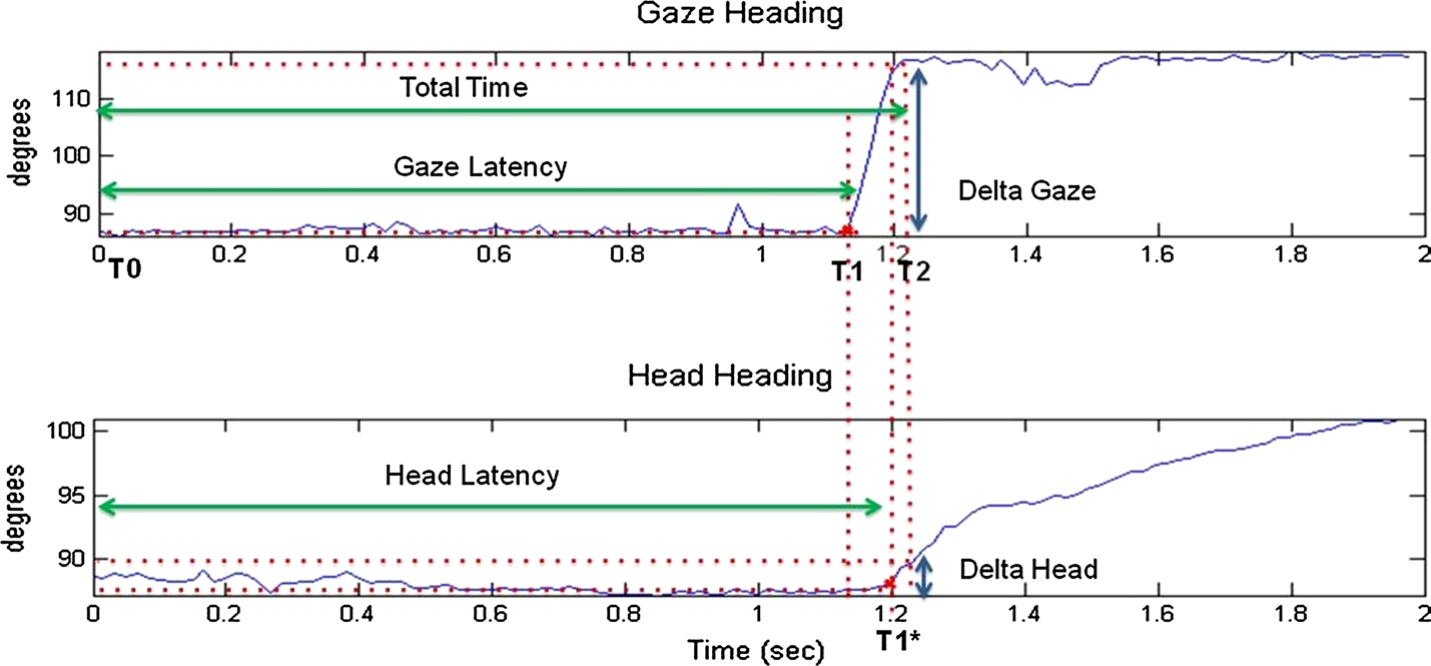
Gaze heading and head heading signal profiles. An example of gaze heading and head heading time response with relative Total Time (TT), Gaze Latency (GL) and Head Latency (HL) parameters

Ideally, the profile of the *Gaze* signal is represented by a ramp divided into three phases (Fig. 4): (a) a fixation phase on the central screen, (b) a gaze movement in the direction of the peripheral screen and (c) a new fixation on the peripheral screen. From this profile, it is possible to identify three main time instants:

- T0 the stimulus appears on the peripheral screen
- T1 the gaze begins to move towards the peripheral screen
- T1* the head begins to move towards the peripheral screen
- T2 the gaze intersects the peripheral screen and the movement is completed

Starting from the previous time instants the following time parameters have been selected:

- T0–T1: *Gaze latency* (*GL*)
- T0–T1*: *Head Latency* (*HL*)
- T1–T2: *Move time* (*MT*)
- T0–T2: *Total time* (*TT*)

In addition, we identified delta parameters related to the angular movements:

- *Delta Gaze* (DG) the angular shift of gaze during MT
- *Delta Head* (DH) the angular shift of head during T1*–T2 interval
- *Delta Eye* (DE) the difference between DG and DH

### Data selection

Starting from a more accurate literature evaluation [28], it is important to take into account several factors influencing data quality: different participants’ characteristics, level of the operator who performs data acquisition, kind of task used during trial sessions, varying environmental conditions, and last but not least, eye tracker specifications, in terms of: cameras resolution, sharpness of the eye images and calibration procedure. We carefully took care of this last aspect in order to prevent a high data loss rate. Thus, at the beginning of each trial session, we carefully performed a double calibration in order to calibrate both the system and the infant’s gaze.

The system calibration was divided into two phases: the first one aimed to adjust the six system cameras to desired positions (small displacements were needed in order to keep the infant’s face centred in at least four cameras) and relative camera brightness and focus. This phase consisted of showing attractive and coloured figures combined with different sound stimuli to the infants, sequentially in the five screens. Then, in the second phase, SmartEye Pro 5.9 application automatically detected the current positions and orientation of the cameras, by moving a little chessboard in front of the cameras to calculate their relative position in respect to the whole setup. Starting from these positions, it calculates head and eyes positions.

The purpose of the gaze calibration was to determine the difference between the visual and the optical axis of the eye. We defined five calibration points corresponding to each centre point of the five screens as objects in the syntax of the 3D world model, and the system automatically created calibration points on each screen. This procedure consisted of showing a smile emoticon growing from the centre of each screen combined with a sound stimulus in order to address the infant’s gaze to the AOI centres. If the calibration was not successful, e.g. one or more calibration point was missing, we repeated the process at least two times to obtain a satisfactory calibration for all five locations.

**Fig. 5:**
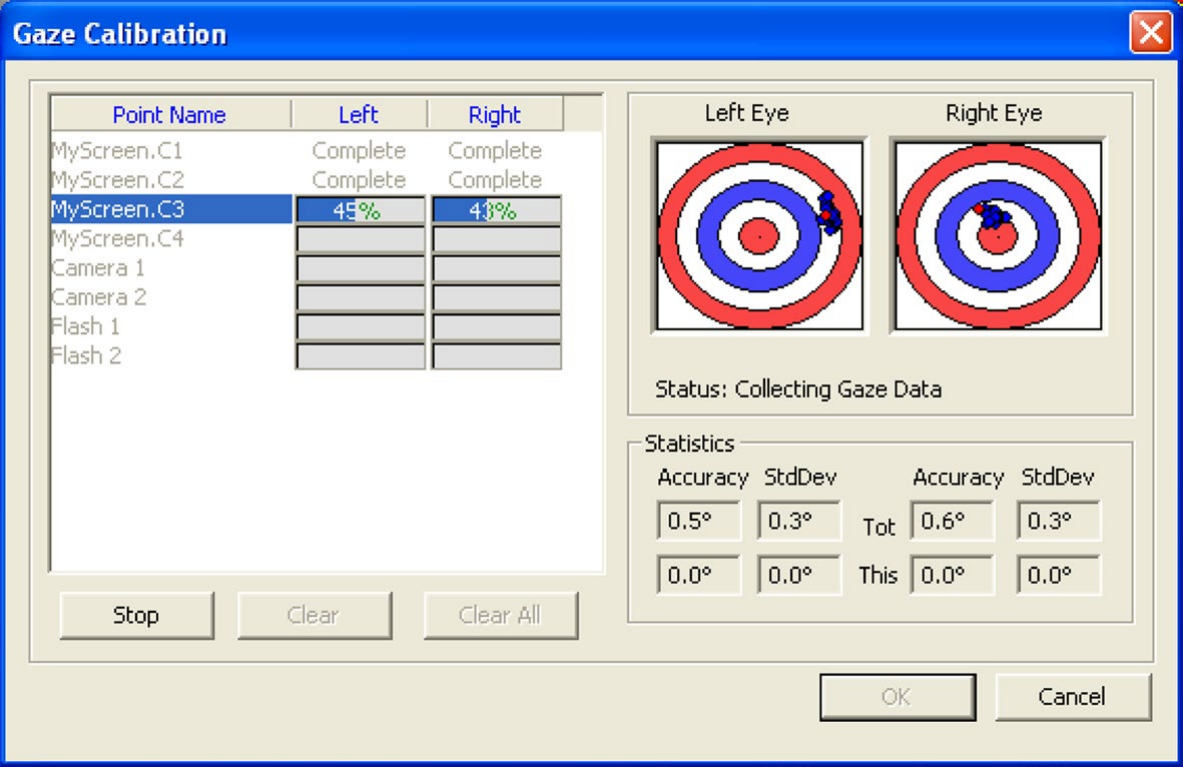
Gaze calibration procedure. The *red dot* shows where the current un-calibrated gaze intersects a plane, orthogonal to the current world point and a vector pointing towards the centre of the eye. The *blue dots* represent all saved samples, whereas the *green dots* show the samples once the calibration algorithm has been run on them. Ideally the *green dots* should be in the middle of both the target. One *circle* in the target corresponds to ±2° of accuracy. We carefully checked that the *blue dots* were close together without outliers, and any outliers that were found were cleared and new samples were added again. We manually repeated this operation until the noise became smaller

Thanks to SmartEye Pro 5.9 application, it was also possible to quantify the accuracy of the calibration points obtained during recording (Fig. 5). For each point we checked the accuracy and standard deviation of the calibration. The accuracy depended on various parameters such as: (a) distance from the cameras, that we assumed remained fixed (60 cm); (b) distance and position of the screens (fixed at 60 cm); (c) individual differences among infants that we did not include in this study [29]. The calibration was repeated until all calibration points had accuracy lower than 1°.

Moreover, regarding data quality and according to SmartEye technical specifications, it was possible to perform a preliminary data quality evaluation thanks to an important system parameter: *Gaze direction quality* that is presented in the range [0.0, 1.0]. When the *Gaze direction quality* is 0, it means that the system is not tracked correctly and loses the infant’s gaze. This parameter expresses a threshold value in order to distinguish reliable data from that to be rejected. Basically it represents a trade-off between recorded data system availability and data accuracy. In order to maintain a reasonable amount of data with sufficient accuracy, we decided that values of gaze direction quality lower than 0.4 would be considered unreliable and consequently rejected.

Furthermore, analysing data in comparison to the gaze heading frame of reference (Fig. 1), we decided to exclude the following cases from the analysis:

- all the trials in which the infant’s gaze did not start from the central stimulus
- all the trials in which the gaze shift was outside the working space (±90°).

### Data analysis

**Fig. 6:**
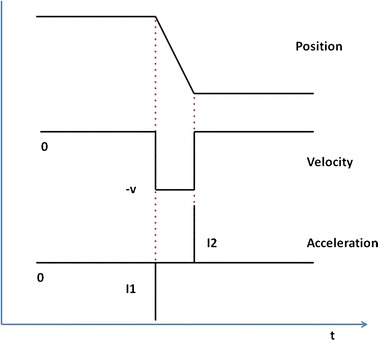
Gaze heading signal, velocity and acceleration profile

Data analysis was devoted to the identification of the time parameters mentioned above and the relative angular movements of head and eyes. T0 can be easily extracted by the time stamp corresponding to the instant in which the stimulus appears on the peripheral screen given by the stimuli management software. The acceleration vector corresponding to *Gaze heading* has been used to find T1. When the gaze begins to move, there is a peak in the acceleration signal and this represents the exact parameter of interest (Fig. 6). The acceleration vector has been obtained starting from *Gaze* vector (second derivative of *Gaze* parameter).

The adopted strategy was to find all the highest points that represent the peaks and relative time coordinates, and rejecting all gaze movements in the wrong direction. The value of the *Gaze heading* at the left of the subject is 0°, 90° at the centre and 180° at the right (see the Fig. 1). If the infant shifted the gaze from screen 3 towards screen 4, we expected a growing gaze angle (for opposite movements, we expected decreasing values). The same procedure was used to find T1* from the head heading vector. T2 can be selected from the SmartEye software. Starting from a 3D representation of the external world, the software returns information about the intersection of the gaze with a screen, so it is possible to exactly determine the time in which the gaze intersects each peripheral screen (T2). The spatial parameters of the angular shift are calculated accordingly.

Clinical data was analysed by means of the Statistical Package for Social Sciences (SPSS, version 20.0). Means and standard deviation were calculated and reported in Table 1.

**Table 1:** Mean values of the main parameters and their relative standard deviation (SD)

	AT		FT		AVT	
	60°	30°	60°	30°	60°	30°
Time						
MT (s)	0.618 ± 0.347	0.072 ± 0.017	0.161 ± 0.036	0.109 ± 0.102	0.196 ± 0.133	0.123 ± 0.165
GL (s)	0.987 ± 0.492	1.026 ± 0.322	1.210 ± 0.687	0.874 ± 0.299	0.548 ± 0.199	0.638 ± 0.241
TT (s)	1.605 ± 0.181	1.099 ± 0.326	1.371 ± 0.683	0.983 ± 0.287	0.744 ± 0.096	0.761 ± 0.109
Delta						
DG (°)	52.24 ± 6.60	27.39 ± 7.08	55.76 ± 4.67	29.58 ± 5.05	58.43 ± 5.66	32.00 ± 9.09
DH (°)	27.72 ± 8.06	5.64 ± 5.25	30.24 ± 14.16	14.84 ± 9.32	27.11 ± 8.68	7.79 ± 6.02
DE (°)	24.51 ± 3.64	21.75 ± 8.36	25.52 ± 14.23	14.74 ± 10.67	31.32 ± 7.20	24.21 ± 4.66

Mann–Whitney U independent sample test was used to analyse the following comparisons in all three tasks: differences between 30° and 60° for each parameter and differences between DE and DH both at 30° and 60°.

The same non-parametric test was used to compare differences between attention and audio-visual tasks (both 30° and 60°) for each time parameter.

## Results

Experimental tests gave important results about the capability of the proposed system to track the gaze during the execution of the experimental paradigms. The application of the criteria described in the data selection paragraph brought about a reduction of the total amount of data with a final data loss of approximately 40 %.

**Fig. 7:**
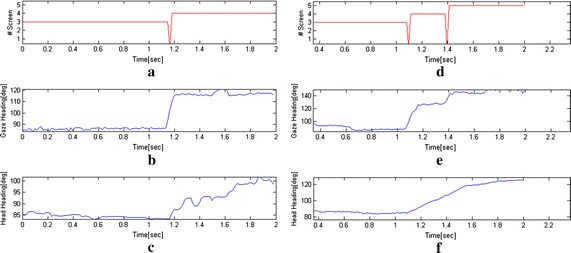
Examples of attention task: **a** results of SmartEye analysis of intersection between the gaze with the screens. During the transition from screen #3 to #4, the gaze passes through the space between the two screens thus it does not intersect one of the AOI and the system returns zero value, **b** gaze heading during the transition from screen #3 to screen #4, **c** head heading during the transition from screen #3 to screen #4, **d** results of SmartEye analysis of intersection between the gaze with the peripheral screens. In this case the system returns zero value when the gaze is between screen 3 and 4 and between 4 and 5, **e** gaze heading during the transition from screen #3 to screen #5, **f** head heading during the transition from screen #3 to screen #5

Figure 7 shows a typical attention task in which an infant orients his/her attention to the periphery (left: from screen 3 to 4, i.e. 30°, right: from screen 3 to 5, i.e. 60°). More specifically, it shows how transition of the gaze from the central screen (#3) to the peripheral one (#4 or #5) works and data about gaze and head position return information about the contribution of head and eye during the required movement.

The proposed system allows to track the gaze and the head in the whole AOI. A good quality of head position is present when the head is found and tracked in at least two cameras of the eye-tracker. Thank to the presence of six cameras, the head is almost visible in two cameras during the entire duration of the tasks. This particular feature reduces the data loss and inaccuracies that might result from nonoptimal head orientations revealed in other works [30].

It is worth to mention also noise issue, the SmartEye system gives the possibility of setting parameters that affects the gaze output values called ‘Filtered’ and consequently the signal noise (e.g. saccade and pupil filters). In details, it is possible to specify at which angle an eye movement will be classified as a saccade and how long the fixation filter should be. In addition, the diameter of the pupil does not change very rapidly; temporal filtering is therefore used successfully to reduce the amount of noise.

From data analysis of the three tasks, it is possible to observe relevant information presented in the following sections about system capabilities to measure infant behaviour in all of the three paradigms. Results are presented on the basis of the defined parameters.

### Time parameters

**Fig. 8:**
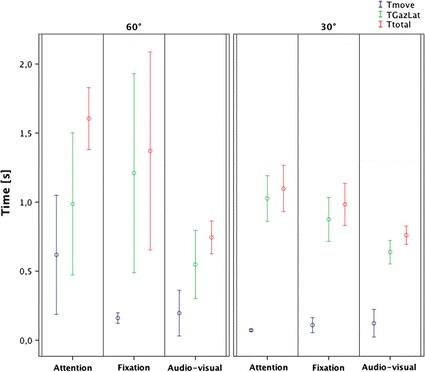
Time parameters results

In all the three tasks, MT values are lower when stimuli are presented at 30° with compared to 60° (AT: p = 0.001, FT: p = 0.003, AVT: p = 0.015) (Fig. 8).

Attention tasks present similar values of GL at 30° and 60° (p > 0.05) as in audio-visual ones (p > 0.05), while in the fixation task it is possible to observe slightly higher and more variable values at 60° compared to 30°(p > 0.05). It is worth saying that 60° values present high variability (Fig. 8).

Since TT is the sum of the previous values, we can observe that in the attention task, TT values are higher at 60° compared to 30° (p = 0.003). The same trend is present in the fixation task even if it is not significant (p > 0.05). This may be due to the high variability of the values at 60°. On the contrary, the audio-visual task presents similar values of TT at 30° and 60° (p > 0.05) (Fig. 8).

### Comparison of time parameters between attention and audio-visual tasks

In the audio-visual task, at 30° TT and GL are largely lower than in the attention (TT: p = 0.016, GL: p = 0.008), while MT presents the same value (p > 0.05). At 60°, TT is largely lower in the audio-visual task (p < 0.009) while the other two parameters are slightly lower (MT: p = 0.047, GL: p > 0.05).

### Delta parameters

**Fig. 9:**
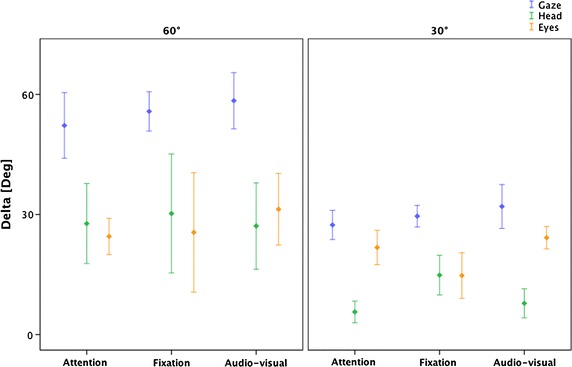
Delta parameters results

By comparing values between 60° and 30°, in all three tasks, DG and DH values at 60° are significantly higher than at 30° (DG: AT p = 0.001, FT p < 0.001, AVT p = 0.003; DH: AT p = 0.001, FT p = 0.008, AVT p = 0.004). DE values are similar in attention and fixation tasks (p > 0.05) and also in the audio-visual even if the difference between the two values are limited (p = 0.04). In the attention and audio-visual tasks, at 30°, it is possible to observe that DE values are higher than DH values (AT: p < 0.001, AVT: p < 0.001) while they are similar in fixation task (p > 0.05). At 60°, DE values and DH values are similar in all the three tasks (p > 0.05) (Fig. 9).

## Discussion

In the last decade there has been an explosion of research using eye tracking with infants thanks to the evolution of the technological solution. However, automatic eye tracking presents several challenges such as the need for a good calibration procedure, the need for a purposeful experimental paradigm for infants and the difficulties of data processing [31].

In this work, the technological challenge was to build a system able to measure an infant’s gaze in a wide visual field covering a total visual range of ±60° from the centre with an intermediate evaluation at ±30°. Moreover, the same system, thanks to different integrated software, was able to provide different visual paradigms (as gap, overlap and multisensory) assessing and comparing different visual sub-competencies. The proposed system endowed the integration of a commercial eye-tracker into a purposive setup in a smart and innovative way. The calibration procedures of the system and the infant’s gaze allowed us to obtain reliable data with an accuracy less than 1°.

One encouraging result is represented by the feasibility of the assessment. The infants performed the three tasks and the system acquired their quantitative data.

The infants maintained the sitting posture with ease and the environment allowed the infant to only pay attention to the stimuli, avoiding the distracting factors. Also the duration of the experiment was well calibrated: all the infants managed to successfully complete all the three tasks.

The results provided two kinds of data: delta and time values. The system allowed us to obtain detailed information of each task about the gaze shift and visual attention in terms of distance and movement of the head and eyes as well as duration and latency. Additionally, it was possible to make comparisons in each task between 30° and 60° and also between tasks (AT vs AUV).

Compared with previous studies, the expectations and the past findings have been obtained, and it has been possible to study the behaviour at two distances from the centre of the visual field: 30 and 60 visual degrees.

Even if the small sample cannot give information and generalisations about visual functioning, some interesting findings are promising for the use of CareToy C in the clinical practice. The main strength is that the same system can measure different abilities in the same infant and across infants within different ages, exploring a wide visual field and distinguishing the eye and the head component contributions to the gaze. An interesting finding about time parameters is that the system was able to discriminate between different behaviour in presence or absence of sound stimuli, confirming the literature [27] that the speed is higher in the presence of sound. In particular, the CareToy C system detected that with the presence of sound the TT values are similar at 30° and 60°. Comparing audio-visual (AVT) and attention (AT) tasks (it is worth underlining that those tasks are identical except for the spatial associations of the visual stimulus with the sound) the system has shown, in the AVT a faster visual response at 30° with lower values of the GL and at 60° lower values not only GL but also of MT. Another interesting result is that the system highlighted higher variability of the TT, related to the GL component in the overlap condition, probably due to the presence of a competitive stimulus (fixation task) which makes the task challenging at 60°. Regarding delta parameters, the analysis of DG confirms the reliability of the CareToy C system because, as expected, the values are in the range of 30° or 60° demonstrating that a larger movement is necessary to reach the more peripheral areas of the visual field. Moreover, the system is able to quantitatively distinguish eye and head components. An interesting finding, to be confirmed in a larger group of infants, is that stimuli at 30° seem to be visually detected using mainly eye movements, while for larger movements (60°) a head compensatory movement is required. This behaviour is evident in attention and audio-visual tasks, instead of fixation task, in which the head contribution is already evident at 30°; this could be due to the presence of a competitive stimulus and the relative difficulty in the gaze disengagement. Further studies on the different strategies at 30° and 60° across different ages could be very interesting for new hypotheses of visual development and applications (e.g. new treatment strategies).

This study presents some limitations. First of all, the data loss remains a critical point; future work will investigate algorithms for improving data quality [32] and the possibility to trigger the stimuli on the screens on the basis of the current position of the gaze obtained with the eye-tracker. Furthermore, the sample size of this study was quite small, but it allows us to demonstrate the feasibility of the purpose. An interesting future development could be to test a wider sample in order to obtain quantitative about the general development of visual perception in infancy.

### Conclusion

In conclusion, this new system is a suitable tool for measuring and evaluating infants’ gaze capabilities in a wide visual field in order to provide quantitative data that can enrich the clinical assessment, e.g. for objectively evaluating changes after a treatment [33].

## Abbreviations

AOI: area of interest
VOG: video-oculography
POG: point of gaze
GUI: Graphical User Interface
LAN: Local Area Network
NTP: Network Time Protocol
AT: attention task
FT: fixation task
AVT: audio-visual task
GL: gaze latency
HL: head latency
MT: move time
TT: total time
DG: Delta Gaze
DH: Delta Head
DE: Delta Eye
